# Extensive Error in the Number of Genes Inferred from Draft Genome Assemblies

**DOI:** 10.1371/journal.pcbi.1003998

**Published:** 2014-12-04

**Authors:** James F. Denton, Jose Lugo-Martinez, Abraham E. Tucker, Daniel R. Schrider, Wesley C. Warren, Matthew W. Hahn

**Affiliations:** 1 School of Informatics and Computing, Indiana University, Bloomington, Indiana; 2 Department of Biology, Indiana University, Bloomington, Indiana; 3 The Genome Institute at Washington University, Washington University School of Medicine, Saint Louis, Missouri; Center for Genomic Regulation, Spain

## Abstract

Current sequencing methods produce large amounts of data, but genome assemblies based on these data are often woefully incomplete. These incomplete and error-filled assemblies result in many annotation errors, especially in the number of genes present in a genome. In this paper we investigate the magnitude of the problem, both in terms of total gene number and the number of copies of genes in specific families. To do this, we compare multiple draft assemblies against higher-quality versions of the same genomes, using several new assemblies of the chicken genome based on both traditional and next-generation sequencing technologies, as well as published draft assemblies of chimpanzee. We find that upwards of 40% of all gene families are inferred to have the wrong number of genes in draft assemblies, and that these incorrect assemblies both add and subtract genes. Using simulated genome assemblies of *Drosophila melanogaster*, we find that the major cause of increased gene numbers in draft genomes is the fragmentation of genes onto multiple individual contigs. Finally, we demonstrate the usefulness of RNA-Seq in improving the gene annotation of draft assemblies, largely by connecting genes that have been fragmented in the assembly process.

## Author Summary

The initial publication of the genome sequence of many plants, animals, and microbes is often accompanied with great fanfare. However, these genomes are almost always first-drafts, with a lot of missing data, many gaps, and many errors in the published sequences. Compounding this problem, the genes identified in draft genome sequences are also affected by incomplete genome assemblies: the number and exact structure of predicted genes may be incorrect. Here we quantify the extent of such errors, by comparing several draft genomes against completed versions of the same sequences. Surprisingly, we find huge numbers of errors in the number of genes predicted from draft assemblies, with more than half of all genes having the wrong number of copies in the draft genomes examined. Our investigation also reveals the major causes of these errors, and further analyses using additional functional data demonstrate that many of the gene predictions can be corrected. The results presented here suggest that many inferences based on published draft genomes may be erroneous, but offer a way forward for future analyses.

## Introduction

Genome comparisons have revealed significant variation in gene family size, both within and between species, e.g. [1]–[7]. This variation can result from either the gain or loss of genes, each of which in turn may be favored by selection. Variation in the number of genes may have important consequences for understanding differences between species, especially for key morphological, physiological, and behavioral traits, e.g. [8], [9], [10].

The observed variation in gene numbers may represent genetic diversity resulting from the evolution of gene families [11], but may also have been incorrectly inferred from sequencing and assembly artifacts. In order to assess the genomic content of a particular species, current methods rely on published genome assemblies. Unfortunately, a major problem in genomics is assembly quality, especially given that it is very difficult to determine the accuracy of *de novo* assemblies [12], [13] and the fact that different assembly algorithms may give very different results [14]. Both computational and experimental methods have been applied to improve upon an assembly: computational approaches include innovations in the assembly algorithms themselves, e.g. [15], as well as methods developed to compare, validate, and gauge the quality of a particular assembly, e.g. [16]–[19]. Experimental approaches have been aimed at improving the connectivity of contigs and scaffolds e.g. [20], assigning and ordering scaffolds on chromosomes, e.g. [21], [22], and validating and refining the annotated genes using RNA data, e.g. [23], [24], [25]. Often computational and experimental methods are used in conjunction to improve an assembly, as further experimental evidence will be integrated or reassembled with the original draft assembly, e.g. [26]. Improvements in sequencing technology do not necessarily mean that assemblies as a whole have improved; indeed, shorter reads have increased the computational complexity of the assembly problem, e.g. [27], [28] and have resulted in more fragmented assemblies (i.e. there are a larger number of contigs). A number of factors confound accurate assembly, including the presence of transposable elements and other repetitive sequences [29], and the allelic variation present when heterozygous individuals are sequenced, e.g. [30]. Despite these obvious problems the number of assemblies produced is increasing, and thousands of genome sequencing projects are planned or in progress [31]. In many cases, gene annotation from the closest annotated relative will be transferred to these new genomes, and will further propagate the annotation problems to many new genome sequences.

**Figure 1:**
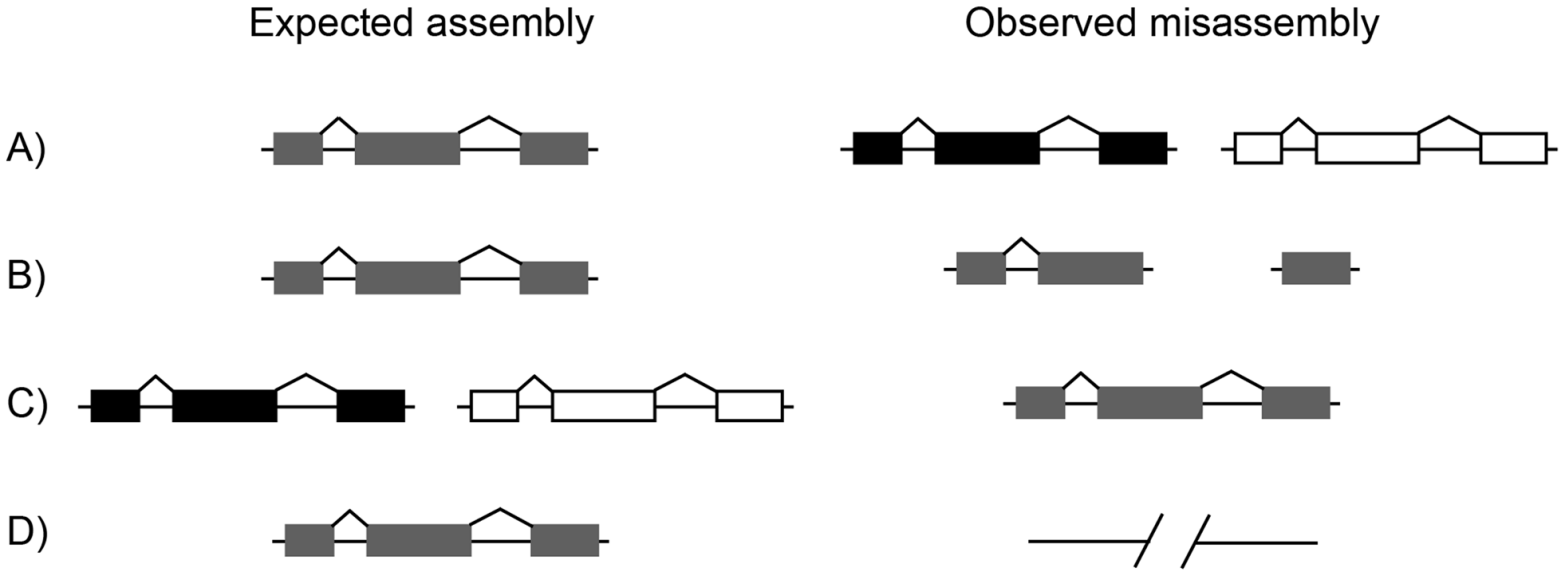
Examples of missassembly leading to misannotation. Each row shows the true state of the genome on the left (“Expected assembly”) and a common misassembly error on the right (“Observed misassembly”). A) A single gene may be assembled as two apparently paralogous loci, increasing the predicted gene count. B) A singe gene may be fragmented into multiple pieces, each on different contigs or scaffolds. This cleavage can increase the number of predicted genes. C) Two paralogous genes may be collapsed into a single gene, decreasing the predicted gene count. D) A gene may be partially or entirely missing from the assembly, decreasing the number of predicted genes.

Low-quality assemblies result in low-quality annotations [18], [27], and these annotation errors cause both the over- and under-estimation of gene numbers, e.g. [32], [33]. One cause of the over-estimation of gene numbers is the splitting of allelic variation (i.e. haplotypes present in heterozygous individuals) into separate loci (Fig. 1A); we refer to such cases as “split” genes. Split genes appear as highly similar duplicated loci within genome assemblies, and are often placed in tandem to one another or with one copy on a small scaffold by itself, e.g. [34], [35]. A second cause of the over-estimation of gene numbers is the fragmentation of a single gene onto multiple contigs or scaffolds (Fig. 1B); we refer to such cases as “cleaved” genes. Because *ab initio* gene predictors less likely to accurately infer gene models across sequence gaps, genes fragmented onto multiple contigs or scaffolds will be predicted as multiple separate genes, e.g. [30]. Note that gene models may also be cleaved simply because *ab initio* predictors have failed to join distant exons together in a single transcript, e.g. [36], [37], though this type of error may be independent of the underlying assembly quality. A common cause of the under-estimation of gene number is the collapse of truly paralogous gene copies into a single locus (Fig. 1C). This occurs because newly formed duplicates are highly similar in sequence, and therefore hard to assemble as separate loci, e.g. [30], [38]. A second cause of under-estimation is simply that genes may not be represented in low-coverage genomes due to a large number of gaps (Fig. 1D). In such cases both total gene numbers and the size of individual gene families may be severely underestimated, e.g. [39].

Many genome assemblies and annotations have improved over time due to further efforts aimed at both increasing sequence contiguity and adding functional data (e.g. RNA-seq) in order to correct gene models. Individual researchers may also contribute to the deconvolution of specific assembly errors, e.g. [27], [40] or to the improvement of specific gene models, e.g. [41], [42]. However, it is often the case that a great deal of research will be based upon the draft assembly before it has reached a finished state, and erroneous conclusions may result, e.g. [40]. As an extreme example, the initial draft human genome contained 223 bacterial genes thought to have been gained by horizontal gene transfer [43]. Closer analysis of this result suggested that many of these cases were simply bacterial contaminants incorrectly assembled into the human genome [44]. As a less extreme example, the initial human genome predicted between 30–40,000 protein-coding genes [43], [45]. As the draft assembly was updated and the gene annotation process was improved, the estimated number of genes in human has continued to fall, and is 20,805 as of February 2014 according to Ensembl [46]. This pattern repeats itself for nearly every draft genome, but is especially true of vertebrate genomes because of their size and complexity [28], [40]. The cascading effects of these errors may affect many downstream conclusions, from inferences about the evolutionary histories of genes to the ability to map genes involved in disease.

Although many consequences of low-quality assemblies have been described, e.g. [27], [28], [47]–[49], few analyses have specifically examined the effect on gene copy-number but see [32], [33]. Because many new, next-generation sequencing technologies are being used to construct genome sequences, we would also like to know the error-characteristics inherent to different platforms. Here we examine gene numbers in multiple genome assemblies, using multiple sequencing technologies, and from multiple species. Our results suggest that low-quality assemblies can result in huge numbers of both added and missing genes, and that most of the additional genes are due to genome fragmentation (“cleaved” gene models). Based on these results we present simulation analyses that suggest that published genomes with surprisingly high numbers of genes may be in error, and further show how these problems can be corrected.

## Results/Discussion

### Errors in *de novo* assemblies of the chicken genome

To determine how total gene numbers are affected by genome assembly quality we compared predicted gene models in multiple versions of the chicken genome. We examined five different assemblies that were based on different sequencing technologies and sequencing depths. These assemblies vary in size and average coverage (Table 1; for more details on these assemblies, see [28]). The 2X fosmid-based assembly (average read length ∼950 bp) may be considered the least complete assembly, as it is the most fragmented, smallest in size, and has the least coverage of the five assemblies considered. The 13X 454-based assembly of the chicken genome was built with 454 single-end reads (average length ∼330 bp), 3 kb mate-pair inserts, and 20 kb mate-pair inserts using the Newbler assembler. The 82X Illumina-based assembly was built with high coverage of paired-end short-insert reads (average length 100 bp) and integrated with inserts of 2 kb in length using the SOAP assembler. The draft chicken reference genome (v2.1) was a 6X Sanger-based assembly that was improved with fosmid and BAC-end sequencing and reassembled with the PCAP assembler (it is also referred to as Galgal3 in some repositories). The final assembly used as a reference, the current chicken reference (v4.0; also referred to as Galgal4 in some repositories), was a further improvement to version 2.1. This hybrid assembly, which was already covered to 6X with Sanger reads, improved to 6.6X with BAC and fosmids, was again reassembled using the following additional 454 sequences: 10X fragment reads, 1.7X 3 kb inserts, and 1.2X 20 kb inserts; again, the PCAP assembler was used to integrate all the data into the final reference assembly. Although it is of high quality, even this reference is considered a “draft” genome.

**Table 1:** Chicken genome assemblies, predicted partial and full-length GENSCAN genes, and completeness of conserved orthologs as assessed by CEGMA.

Assembly	Coverage	Contigs	Partial genes	Full-length genes	Completeness
Fosmids	2X	281711	138354	21250	14.1%
454	12X	45554	73262	36210	68.2%
Ref 2.1	6.6X	71609	86543	38199	66.5%
Illumina	82X	27093	64552	33324	74.6%
Ref 4.0	12X	25017	61405	35537	80.7%

We predicted genes on each of these five assemblies using the *ab initio* prediction methods implemented in GENSCAN [50] and Fgenesh [51]. GENSCAN was used with the “eukaryotic” model specified, and Fgenesh was used with the specific model for chicken available in the package. GENSCAN (Table 1) found a greater number of genes than Fgenesh (S1 Table), which typically produced more conservative counts but also more complete gene models. Both gene predictors found tens of thousands of genes for each assembly, and we found that the assemblies with the most scaffolds also had the most predicted genes (Table 1). However, a great many of the predicted genes (often more than 50%; Table 1) were lacking either a start or stop codon, or both. We suspected that the enrichment of small scaffolds was increasing the number of incomplete predictions, and filtered very small scaffolds (<1000 bp) from the assemblies. This decreased the total number of predictions while also providing a greater proportion of complete gene models. We then extracted only complete gene models—those with both start and stop codons—from each set of predictions. This yielded a similar number of predictions (∼36,000) for all but one of the assemblies. That particular assembly was built solely from fosmids and plasmids: it has an average genome coverage of only 2X and is missing roughly 150 Mb relative to the other assemblies; we were only able to extract 20,000 complete genes from the predictions on this assembly. The fosmid assembly also has the most total predicted genes (when including those without both start and stop codons) as well as the most scaffolds, though both genes and scaffolds were shorter on average than in the other chicken assemblies.

As an alternative method to assess assembly quality, we applied the CEGMA pipeline [52]. CEGMA maps a set of core eukaryotic genes to assemblies in order to determine their completeness—that is, how many of them are represented as full-length gene models [53]. This method has been used by the Assemblathon [14] as one measure of the quality of different genome assemblies. Table 2 reports the completeness of CEGMA genes in each of the chicken assemblies analyzed here. The most up-to-date assembly (v4.0) shows the highest percentage of full-length CEGMA genes (80.7%), while the fosmid-only assembly shows the lowest (14.1%). As the average gene length in the current chicken annotation is 27.8 kb, it is clear that many genes have large pieces missing or are fragmented onto multiple contigs in these assemblies.

**Table 2:** Number of predicted genes in simulated *D. melanogaster* assemblies.

	Number of scaffolds								
Annotation software	707	2164	5225	5730	6296	10674	12354	14061	17941
GENSCAN	22679	23654	25413	25413	26370	28328	29225	30073	32025
Fgenesh	17718	18152	18905	18959	19193	19978	20285	21469	24922
AUGUSTUS	14098	14479	15095	15222	15391	16051	16436	17490	20654
MAKER	12687	13872	14931	15761	16059	16903	18231	21340	23916

**Figure 2:**
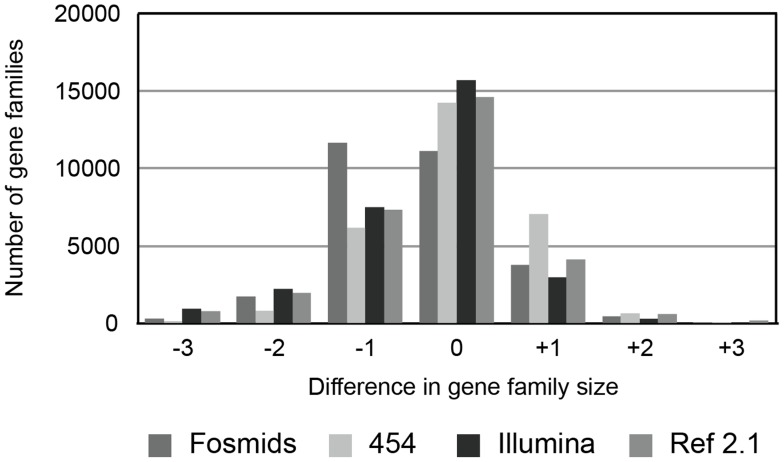
Differences in gene family size when comparing annotated draft genomes (see Table 1 for individual descriptions) to the chicken reference assembly (v4.0). For each gene family, the size (in total number of genes predicted) was compared to the chicken reference; positive numbers indicate an excess number of genes in the draft genome annotations, while negative numbers indicate a deficit of genes. The small number of gene families with more than +/−3 differences from the reference are not shown. Gene models were predicted using GENSCAN.

After clustering the filtered predictions into groups of homologous genes based on sequence similarity (equivalent to gene families; see Methods), we were able to compare gene family sizes in each assembly relative to the predicted sizes in the current chicken reference assembly (Fig. 2). As expected based on quality and coverage, the fosmid assembly shows the largest deviation in terms of gene family size relative to the reference chicken assembly. For each assembly no more than 60% of all gene families were the same size as in the reference assembly, meaning that the remaining 40% or more of families were inferred to have the wrong size. These gene families were either missing one or more genes relative to the reference or contained one or more additional members relative to the size of gene families inferred from the reference assembly. The fosmid assembly was a clear outlier, with more than half of all gene families missing gene copies relative to the reference.

Overall, these results show that different next-generation sequencing technologies have produced assemblies of largely equal quality in terms of gene copy-number, though of course these assemblies have very different coverage levels. For all non-reference assemblies, a huge number of gene families have an incorrect number of copies (assuming that the current reference is correct), which will lead to incorrect inferences about rates of gene family evolution, and false inferences of specific gene gains and losses.

### Examining the cause of errors in a draft chimpanzee genome

We performed a similar analysis on the chimpanzee genome, comparing the original chimpanzee annotation (Pan_troglodytes-1.0) with an updated version of the same genome (Pan_troglodytes-2.1). This analysis differs from the chicken analysis in that we relied solely on the published annotations, and therefore improvements to the predicted gene set may be due to improvements to the assembly, improvements to the *ab initio* gene predictors, and/or additional functional data. However, this analysis also removes the gene-prediction step from our hands, allowing us to evaluate predictions done by the Ensembl pipeline on two different assemblies.

**Figure 3:**
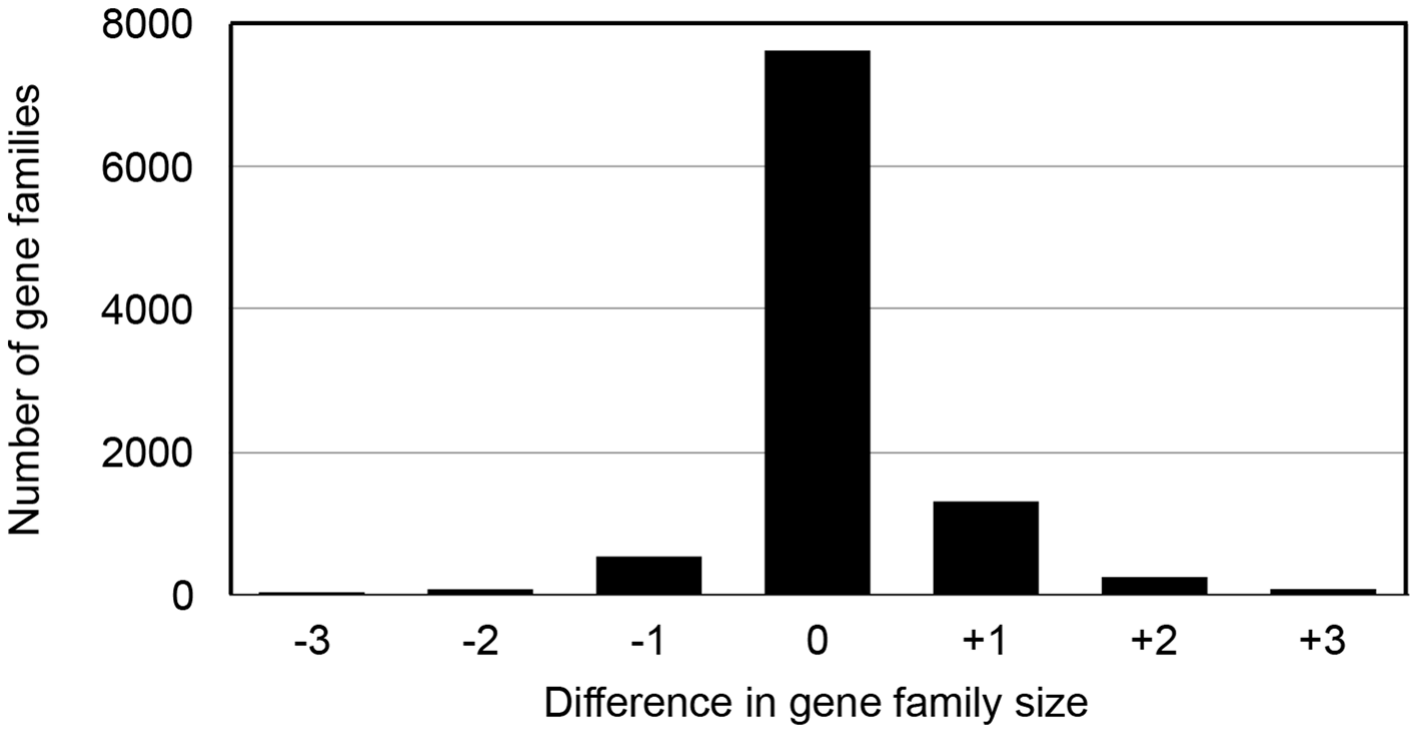
Differences in gene family size when comparing the initial chimpanzee assembly (Pan_troglodytes-1.0) to an updated version (Pan_troglodytes-2.1). Positive numbers indicate an excess number of genes in v1.0, while negative numbers indicate an excess. The small number of gene families with more than +/−3 differences from the reference are not shown.

We find a similar result in chimpanzee as to that found in chicken, with a large proportion of the gene families having incorrect estimates for the number of genes (Fig. 3). Overall, 74% of families had the same number of genes in the two annotations, while 26% had either a greater or smaller number of genes. A major difference between the chicken analysis and the results found for chimpanzee is that the most common error in the draft chimpanzee genome was the addition of a single gene rather than the loss. The earlier assembly and annotation had predicted almost 1,800 more genes than the updated assembly and annotation.

In order to determine the cause of these additions we asked whether the genes in the earlier assembly version were full-length copies of each other (indicative of split alleles; Fig. 1A) or were instead made up of two non-overlapping fragments of the full-length gene found in the updated assembly (indicative of cleaved genes; Fig. 1B). We were able to determine the cause of the additional gene in 1,693 families (Methods). Of these, 1,279 were cleaved genes and 414 were split alleles. This was an unexpected result as the donor chimpanzee, Clint, was heterozygous for over 1 million SNPs [54] and we therefore expected many split alleles; however, the genome also had many gaps, effectively fragmenting it into a large number of pieces.

### Does fragmentation of assemblies lead to higher gene numbers?

Our results from chimpanzee and chicken suggest that the fragmentation of genomes into thousands of contigs may play a large role in falsely increasing predicted gene numbers. Such assembly fragmentation may have played a part in the extremely large number of genes predicted in several published genomes. For example, the crustacean, *Daphnia pulex*, has 30,907 predicted genes [55]. However, the first draft assembly available for this species is extremely fragmented, with a very low N50 scaffold size (<400 kb), over 5,000 scaffolds, and over 19,000 contigs [55]. We suspected that the fragmented nature of the draft assembly, in conjunction with the lack of a high-quality genome annotation from a closely related species, was inflating the gene counts. To indirectly test this hypothesis—and to directly examine the effect of fragmentation on predicted gene numbers—we developed a method to produce increasingly fragmented assemblies of the high-quality *Drosophila melanogaster* genome (Methods). Comparing the genes predicted from these simulated assemblies should reveal the effect of fragmentation.

**Figure 4:**
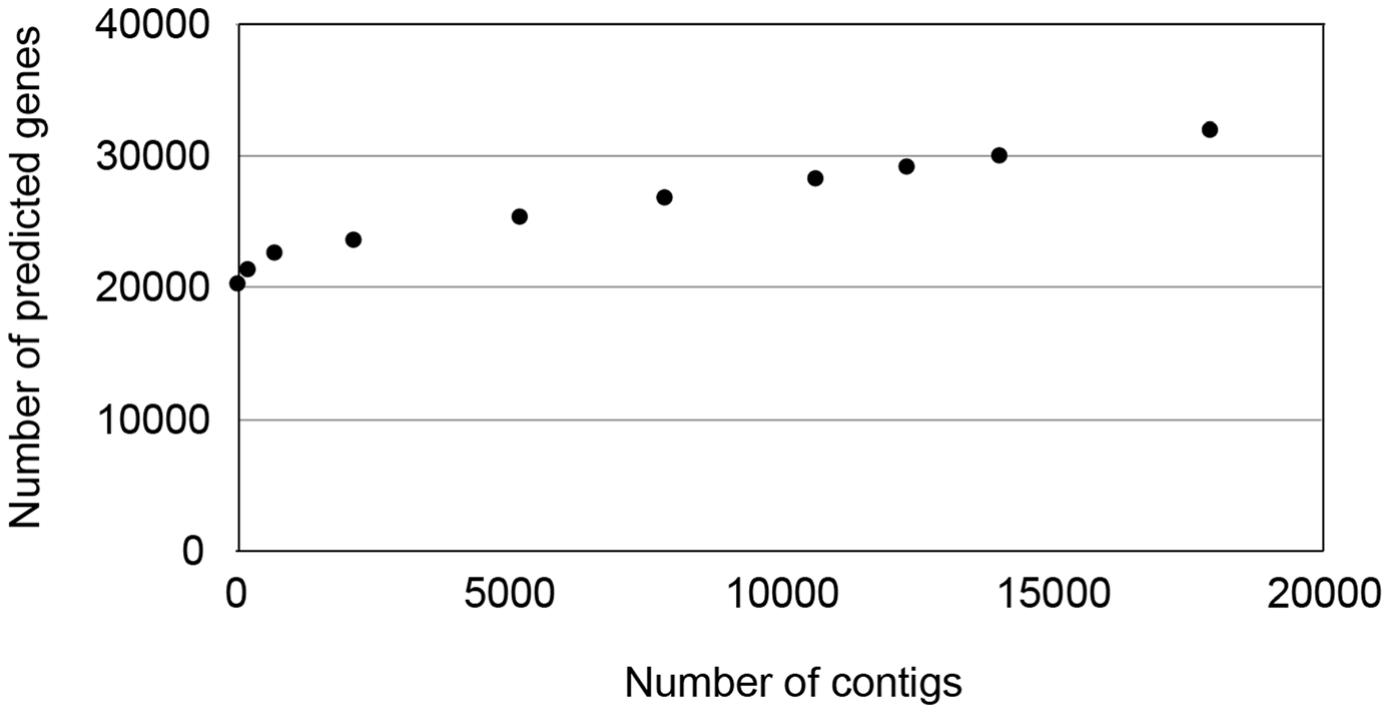
Number of predicted genes increases with increasing genome fragmentation. Starting with the *D. melanogaster* reference genome (release 5.41), the sequence was cut into increasing numbers of “contigs.” GENSCAN gene predictions for each assembly are shown.

We produced nine simulated *D. melanogaster* assemblies with between 707 and 17,941 contigs, and compared the number of predicted gene models in each. We again applied the GENSCAN and Fgenesh gene predictors, as well as the AUGUSTUS predictor [56] and the MAKER gene prediction pipeline [57]. As expected if fragmentation is a cause of increased gene number, the number of predicted genes in each simulated *D. melanogaster* assembly increased as the genomes become more fragmented (Table 2). Strikingly, in the simulated genome with 17,941 contigs—each of which has a length drawn from the distribution of contig lengths in the *Daphnia pulex* genome (Methods)—we find 32,025 GENSCAN-predicted genes with start and stop codons, a handful more than are present in the published *Daphnia pulex* genome (Fig. 4). Although the other predictors all give more modest increases in gene number with increasing fragmentation, they all predict 6,000–10,000 additional genes on the most fragmented assemblies (Table 2).

**Figure 5:**
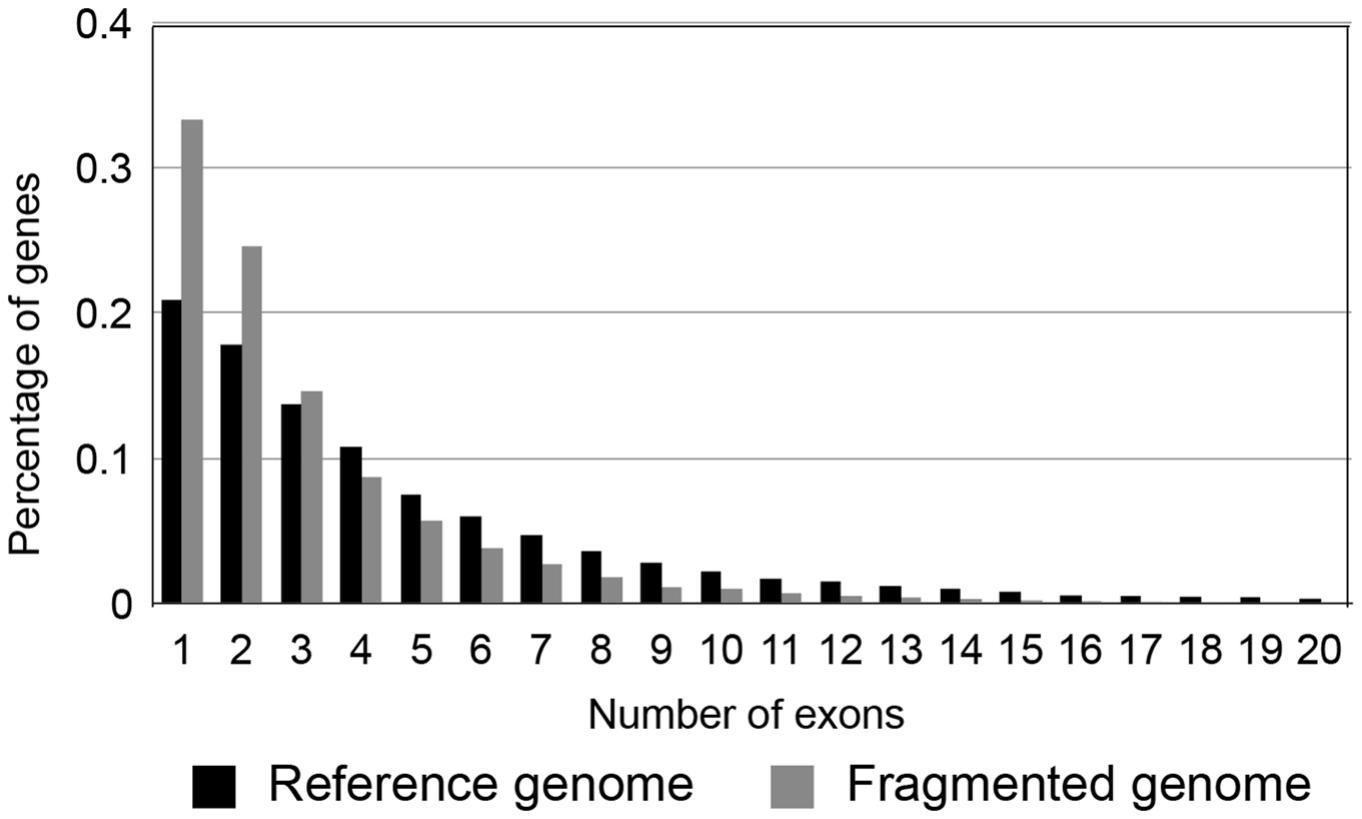
Number of predicted exons per gene decreases with increased genome fragmentation. A comparison of the number of predicted exons per gene in the uncut *D. melanogaster* reference genome to the fragmented version of this genome that contains 17,941 contigs (the right-most point in Fig. 4). Gene models were predicted using GENSCAN.

When we examined specific genes in our prediction sets we often found them to be cleaved, sometimes into multiple pieces, with single exons or groups of exons isolated on individual contigs. Gene prediction software will often call these exons as genes, and the process of gene prediction in these highly fragmented genomes has essentially become one of exon prediction. Zhang *et al.* [40] found similar instances of spurious gene calls from cleaved or isolated exons when looking at the draft rhesus macaque assembly and annotation (see [58] for examples from the pig genome). Although these random cleavages of the *Drosophila* genome may not be a perfect approximation of fragmentation in real assemblies, the increasing fragmentation causes the number of exons per gene in the predicted sets to decline. Comparing the number of exons per gene in the simulated dataset to the number in the reference *D. melanogaster* genome, we see a huge enrichment for single-exon genes and a general decline in the average number of exons (Fig. 5). Due to the highly fragmented nature of this assembly almost none of the genes with over a dozen exons have remained full-length, and the longest genes have often been cleaved into more than two predicted genes.

While the results of our simulated genomes do not directly demonstrate the causes of the over-prediction of genes in published genomes, they do strongly indicate that genome fragmentation can play an outsized role in this problem. However, although many new genomes are highly fragmented, most do not have such large numbers of predicted genes. So why are there differences in predicted gene numbers? For many newly sequenced genomes, high-quality genomes from closely related species can be used to improve the assembly [59], [60], or to directly improve gene models [61]. In the case of *Daphnia pulex* there are no closely related complete genomes, and therefore little comparative data for improvement; as expected from severe fragmentation, 22% of annotated *Daphnia* genes do not have both a start and stop codon. Other data and methods can be used to improve gene annotations, however, and in the next section we show how one such method can be utilized.

### Improving fragmented assemblies using RNA-seq

In addition to data from closely related species and genomes, RNA-seq data (or any kind of transcript sequencing data) has been used to improve both genome assemblies [62], [63] and gene annotations, e.g. [23], [24], [25]. RNA-seq offers an effective method for improving an annotation set, especially when a single gene may span multiple contigs [24]. The sequencing of mRNAs is equivalent to sequencing reads with an insert size of the order of intron sizes—because these regions are removed from mRNAs, even large gaps can be crossed if they coincide with introns. In terms of fragmented genome assemblies, the sequencing of mRNAs allows genes cleaved onto multiple contigs to be identified as a single locus, and therefore for inflated gene numbers to be reduced. While gene models from related species offer an alternative method for identifying fragmented genes [61], RNA-seq can be used for any species, regardless of whether there is a genome with a high-quality annotation that is closely related.

To determine the effectiveness of RNA-seq data in restoring fragmented gene models we obtained reads from 11 published experiments in *D. melanogaster* (Table 2). After mapping paired-end reads from these experiments back to our simulated assembly with 17,941 contigs, we asked whether there were any cases in which two different predicted gene models were uniquely hit by a connected pair of reads. In other words, we looked for pairs of reads for which one hit one predicted gene and the other read hit another predicted gene on a different contig. Even with a single RNA-seq experiment, thousands of predicted genes could be linked by paired-end evidence (Table 3). Although on average only 2% of paired-ends per experiment met our conditions for connecting genes on different contigs, this small percentage represents hundreds of thousands of total connections. As more RNA-seq datasets were analyzed, many of the same connected exons were identified, but each new dataset also added a significant number of novel connections (Table 3; this analysis was only carried out once, with individual datasets added in a random order).

**Table 3:** RNA-seq reads mapped to a simulated *Drosophila* assembly allows correction of the original 32,025 predicted GENSCAN gene models.

No. of datasets	No. of reads	Revised gene count	Reduction	Cumulative reduction
1	6.93E+06	27064	4961	4961
2	1.64E+07	24769	2295	7256
3	2.33E+07	23511	1258	8514
4	3.06E+07	22645	866	9380
5	3.94E+07	21895	750	10130
6	4.86E+07	21413	482	10612
7	5.54E+07	21113	300	10912
8	6.22E+07	20974	139	11051
9	7.06E+07	20853	121	11172
10	8.01E+07	20467	386	11558
11	1.06E+08	20094	373	11931

If we require only a single RNA-seq read as evidence of connected exons, almost 12,000 predicted genes were removed by combining them with other genes, and the remaining estimate of ∼20,000 predicted genes closely resembles the number predicted from the uncut *D. melanogaster* reference genome (Fig. 4). Increasing the number of reads required to connect exons before considering them to be in the same gene resulted in a linear decrease in the number corrected (Table 4). This is to be expected, as even a very large RNA-seq dataset may not have many reads covering the same exon-exon junction; however, increasing the number of required reads may also increase accuracy of inferences [63]. These results demonstrate that RNA-seq can be used effectively to improve gene annotations in highly fragmented genomes. This result is in contrast to the use of microarrays in improving gene annotations, as arrays will only establish that predicted exons are parts of genes, and not unique genes themselves, cf. [55]. It must also be noted that RNA-seq cannot help to improve cases of split alleles (Fig. 1A)—in these cases both of the predicted gene models will be full-length, and the RNA-seq data will not contain any information about the nature of the misassembly.

**Table 4:** Number of GENSCAN gene models connected through RNA-seq alignments, with increasing requirements for the number of connecting reads.

Number of Reads	Number of Models	Collapsed
1	20094	11931
2	23987	8038
3	25492	6533
4	26775	5250
5	27844	4181
6	28745	3280
7	29481	2544
8	29992	2033
9	30493	1532
10	30845	1180

### Conclusions

Our results suggest that low-quality assemblies may contain a great many added or missing genes, especially as cleavage and separation of genes across multiple contigs hinders the ability of gene predictors to correctly identify genes. We have shown that most additional genes are due to such cleavage, and specific cases examined in the chicken and chimpanzee genomes support this finding. Our simulation analyses of fragmented *Drosophila* assemblies indicate that published genomes with surprisingly high numbers of genes may be in error, and simply a result of severe fragmentation. Finally, we have found that RNA-seq offers the ability to correct annotation errors that result from such fragmentation, as fragmented predicted genes can be collapsed with paired end information.

## Methods

### Analysis of the chicken genome

Four chicken assemblies of varying quality (Table 1) were obtained from The Genome Institute at Washington University; they are partially described in [28], [64]. A fifth chicken assembly, the current reference genome (v4.0), was obtained from Ensembl [46]. For each of these assemblies, we first filtered out short scaffolds (<1000 bp) before predicting genes using GENSCAN and Fgenesh. We extracted all predicted genes that were considered complete: that is, their sequence contained both a start and stop codon. After using BLAST [65] to compare all GENSCAN genes from all assemblies to one another, the graph clustering algorithm MCL [66], [67] was used with default parameters to cluster genes into gene families based on these similarity scores. The 29,763 gene families resulting from this procedure contained differing numbers of genes from each assembly, including from the current reference assembly. For each gene family the number of genes in each assembly was compared to the number in the reference chicken assembly.

### Examining the cause of errors in a draft chimpanzee genome

Two assemblies and annotations of chimpanzee, Pan_troglodytes-1.0 and an updated version of the same genome, Pan_troglodytes-2.1, were obtained from Ensembl (versions 35 and 56, respectively). The first version was sequenced to 4X using the PCAP assembler [54]; the second version represents an additional 2X coverage from plasmid reads, and reassembly using PCAP. Following the methods described above for chicken, but with the annotated gene models from Ensembl, we again clustered genes from both assemblies into 11,959 gene families. For all families with a larger number of members in the low-coverage assembly and annotation, we used BLAST to search full-length gene models from the high-coverage against the predicted set of genes in the family. In order to classify genes as “cleaved” we required that there be at least two complementary gene models in the low-coverage set. That is, we required that genes in the low-coverage annotation be non-overlapping, but to match complementary parts of the full-length models. Multiple genes from the low-coverage annotation that matched both the full-length gene model and each other (i.e. were overlapping with >95% similarity over 80% of their length) were classified as “allelic splits.”

### Generating simulated *Drosophila* assemblies

We attempted to transform the high-quality, near-complete *D. melanogaster* assembly into one resembling the *Daphnia pulex* assembly. In order to do this, we first collected information about the *Daphnia pulex* assembly from wFleabase ([68], http://wfleabase.org/), specifically, the scaffold lengths as well as positions and lengths of all gaps within those scaffolds. This filtered scaffold set contained 5,191 scaffolds [68]. However, when we examined the assembled scaffolds we found that nearly 25% of bases were gaps, represented by stretches of N's in the sequence. To understand how gene prediction software would handle such gaps, we manually inserted stretches of N's into the sequence of known *D. melanogaster* genes, and then predicted genes on the artificially created sequence. We found a limitation in the length of a gap that the gene prediction software could span and still predict a single gene. GENSCAN, for instance, could not predict a single full-length gene across a gap of length 50 or greater. This implies that individual contigs are the fundamental unit useful for predicting genes, and that even individual large scaffolds fragmented into many contigs may result in the over-prediction of genes. We therefore chose 50 bp as a minimum cutoff length for the length of gaps, separating scaffolds into individual contigs when stretches of N's longer than fifty characters were found. Applying this cutoff to the *Daphnia pulex* assembly revealed 17,924 “contigs” useful for gene prediction.

*Drosophila melanogaster* assembly release 5.44 was obtained from Flybase [69], in the form of six chromosome files. Using the distribution of contig sizes found in the *Daphnia pulex* assembly, we generated 10 simulated *D. melanogaster* assemblies with different numbers of contigs (Table 4). To do this, for any specified number, *x*, of contigs needed for the simulated *D. melanogaster* genome we took the longest *x* contigs from the *Daphnia pulex* assembly. The reference *D. melanogaster* genome was then fragmented into *x* pieces by randomly cutting contigs of the lengths drawn from the *Daphnia pulex* assembly, while ensuring that the entire *D. melanogaster* sequence was included in each simulated dataset. Because the *Daphnia pulex* genome is roughly 170 Mb in length (not including N's) while the *D. melanogaster* genome is 138 Mb, we are conservatively excluding the class of extremely small scaffolds found in *Daphnia pulex* from our simulated genomes. We predicted genes on each simulated assembly using GENSCAN, Fgenesh, AUGUSTUS, and MAKER. Although GENSCAN was used with a pre-specified human model, this has been shown to be sufficient for most eukaryotes e.g. [51]. Fgenesh has a specific *Drosophila* model, and as a consequence produced much lower gene counts.

### RNA-seq analysis

Paired-end RNA-seq data from an experiment by the Berkeley Drosophila Genome Project [70], was obtained from the public database ENA ([71], http://www.ebi.ac.uk/ena/). These paired end reads were mapped against the simulated *D. melanogaster* assembly that had ∼18,000 contigs using the software BWA [72] with default parameters. Additional processing of the alignment was performed using samtools [73]. We filtered by read quality and mapping quality, and sought connecting paired-end reads where each end mapped to different scaffold. We used the positions of every exon in the predicted gene set for our simulated assembly to determine which exons were associated by the connecting paired-end reads. A set-merging algorithm was applied to chain together connected exons before the resulting gene set was analyzed.

## Supporting Information

S1 Table Assembly statistics and gene models predicted by Fgenesh for chicken genome assemblies.
(DOCX)
