# Supplementary material for: Extensive Error in the Number of Genes Inferred from Draft Genome Assemblies
Source: PLoS Comput Biol. 2014 Dec 4;10(12):e1003998. doi: 10.1371/journal.pcbi.1003998 (PMC4256071; doi:10.1371/journal.pcbi.1003998)
Supplement: S1 Table — Assembly statistics and gene models predicted by Fgenesh for chicken genome assemblies. (DOCX) [file pcbi.1003998.s001.docx]

S1 Table: Assembly statistics and gene models predicted by FGENESH for various chicken genome assemblies, with contigs less than 1 kb removed.

| Assembly | Technology | N50 length | Number of n50 contigs | Total assembly length | Total FGENESH gene models | Complete FGENESH gene models |
| --- | --- | --- | --- | --- | --- | --- |
| Fosmids | 1.8x plasmids, .36x fosmids; PCAP | 3193 | 66072 | 7.89E+08 | 55678 | 17638 |
| 454 | 11x fragments, 1x 3 kb, .78x 20 kb; Newbler | 550052 | 514 | 1.12E+09 | 53860 | 28035 |
| Ref 2.1 | 6X Sanger plasmids, fosmids, BAC ends; PCAP | 45875 | 5819 | 1.04E+09 | 59344 | 28971 |
| Illumina | 68x short inserts, 14x 2 kb; Soap | 344055 | 743 | 9.90E+08 | 49718 | 25319 |
| Ref 4.0 | 10X Titanium, 1.7x 3 kb, 1.2X 20 kb; PCAP | 248582 | 1099 | 9.41E+08 | 69651 | 32888 |
